# A qualitative assessment of direct-labeled cDNA products prior to microarray analysis

**DOI:** 10.1186/1471-2164-6-36

**Published:** 2005-03-11

**Authors:** Sherry F Grissom, Edward K Lobenhofer, Charles J Tucker

**Affiliations:** 1 National Institute of Environmental Health Sciences Intramural Microarray Group, Research Triangle Park, NC 27709, USA; 2 Gene Regulation Group, Laboratory of Molecular Carcinogenesis, National Institute of Environmental Health Sciences, Research Triangle Park, NC 27709, USA; 3 Paradigm Array Labs, Icoria, Inc., Research Triangle Park, NC 27709, USA

## Abstract

**Background:**

The success of the microarray process in determining differential gene expression of thousands of genes is dependent upon the quality and integrity of the starting RNA, this being particularly true of direct labeling via a reverse transcription procedure. Furthermore, an RNA of reasonable quality still may not yield reliable hybridization data if the labeling efficiency was poor.

**Results:**

Here we present a novel assay for assessing the quality of directly labeled fluorescent cDNA prior to microarray hybridization utilizing the Agilent 2100 Bioanalyzer, which employs microfluidic technology for the analysis of nucleic acids and proteins. Using varying amounts of RNase to simulate RNA degradation, we show the strength of this un-advertised assay in determining the relative amounts of cDNA obtained from a direct labeling reaction.

**Conclusion:**

Utilization of this method in the lab will help to prevent the costly mistake of hybridizing poor quality direct labeled products to expensive arrays.

## Background

The use of cDNA and oligonucleotide microarray technology has revolutionized the fields of molecular biology, biochemistry and genetics. The ability to simultaneously evaluate gene expression across tens of thousands of genes gives researchers opportunities not previously afforded to them.

RNA extractions have proven to be of large concern for evaluating messenger RNA transcript levels by microarrays and other procedures such as RT-PCR, RNase protection assays and Northern blot analyses. Extraction procedures are still evolving and adapt to meet different needs, such as for pure cell populations [1]. Differences between two RNA extractions from the same source material have been shown to make a significant contribution to technical variance in microarray data [2].

Microarray technology utilizes various protocols based in part on reverse transcription and PCR technologies [3,4]. Direct labeling protocols use modified deoxyribonucleotide phosphates (dNTPs) incorporated during a reverse transcription reaction, in which mRNA is copied into cDNA. One possible modification to the dNTPs is the addition of an amino-propagyl cyanine (Cy) fluorescent molecule at the 5-carbon of the pyrimidine base [5,6]. For cDNA and oligonucleotide microarrays, Cy3 and Cy5 are commonly used fluorescent dyes that are excited by different wavelengths of light. Therefore they can be used in combination, one labeling a control or reference sample and the other labeling the treatment or test sample. After combining the two labeled cDNA samples and hybridizing to a microarray chip, gene expression can be extrapolated from the ratio of the two different cyanine dye fluorescences detected. The entire microarray process, especially the reverse transcription labeling procedure, is dependent upon the quality and integrity of the starting RNA.

The Agilent 2100 Bioanalyzer, first described for the investigation of DNA, employs microfluidic technology for the analysis of nucleic acids and proteins [7]. A total RNA assay determines a numerical value for the ratio of 28S ribosomal RNA (rRNA) subunit to 18S rRNA subunit, while an mRNA assay determines the percentage of rRNA contamination. To measure ribosomal subunit concentrations, RNA is combined with a sample buffer containing a fluorescent dye that intercalates into the RNA and is excited by an internal 635 nm diode laser. Data output is in the form of an electropherogram, which graphically depicts spikes in fluorescence over time; the larger the peak, the more intact ribosomal subunits are in the sample. If a sample is degraded, subunits will show a smaller degree of fluorescence that is spread out over a longer amount of time, indicating a greater variety of sizes in the sample. Auer and colleagues recently published the "degradation factor" utilizing data obtained using the RNA assay of the Bioanalyzer as a more quantitative approach [8]. This approach calculates a ratio between the 18S ribosomal peak area and the average of the peaks smaller than the 18S ribosomal peak that are indicative of degradation. The authors show that RNA isolations which exhibit similar degradation factors are more likely to give gene expression results that are more biologically relevant than comparing two RNA isolations with differing degrees of degradation.

The quality and integrity of RNA samples can be evaluated by gel electrophoresis, UV spectrophotometry and the Agilent Bioanalyzer. From one or a combination of these methods, assessments of how well an RNA sample may perform in a reverse transcription labeling reaction and subsequent microarray chip hybridization can be made. Unfortunately, the cDNA reverse-transcribed from an RNA of reasonable quality may not yield reliable products after the labeling steps and lead to poor hybridization results. Our lab has experienced this problem as a result of, among others, possible genomic DNA contamination of the RNA sample. This problem with the RNA is difficult to detect with an RNA assay on the Bioanalyzer, and the contamination will likely impact the quality of any labeling reaction the sample is used in, regardless of the fluorophore.

Agilent currently promotes the use of their instrument for measuring Cy-labeled cRNA obtained from an amplification protocol. They do not, however, currently have a protocol to measure the quality of Cy-labeled cDNA from a direct labeling protocol. To address and avoid the loss of time and money associated with a failed microarray analysis, we present a novel use for the Agilent 2100 Bioanalyzer in determining the relative quantity and quality of direct-labeled products obtained during the labeling reaction. By comparing with cDNA obtained from known high quality RNA, we can determine how well the cyanine dye was directly incorporated during reverse transcription, and thus if reliable microarray data can be obtained.

## Results and discussion

**Figure 1:**
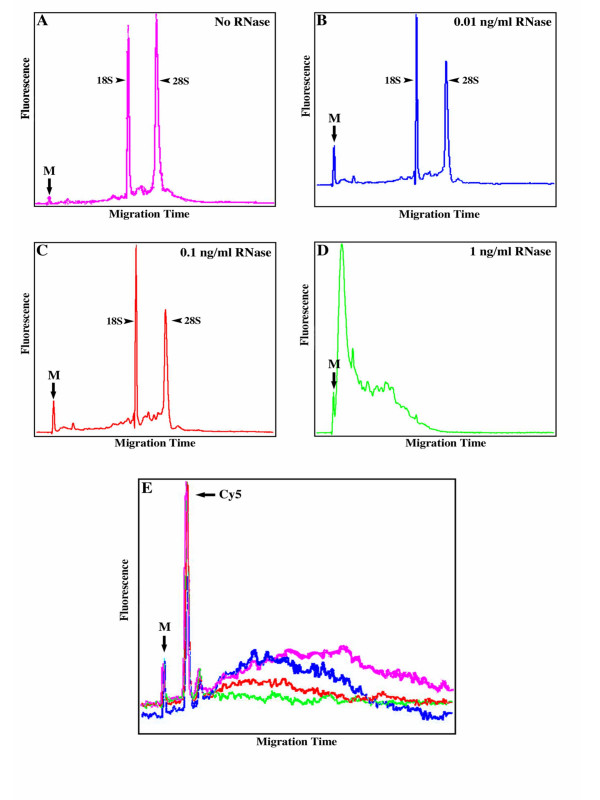
**RNA and Cy5-dUTP labeled cDNA measured by the Agilent 2100 Bioanalyzer. **Electropherogram images (one representative experiment) of RNAs treated with RNase as follows: No RNase (A, pink), 0.01 ng ml-1 (B, blue), 0.1 ng ml-1 (C, red), 1 ng ml-1 (D, green). Overlayed electropherogram image of Cy5-dUTP labeled cDNA (E, colors as described above, one representative experiment). The first peak (as shown by an arrow) from the left in all electropherograms is a 50 base pair marker present in the sample buffer (M). The second peak (as shown by an arrow) that is present only in Cy5 samples is free Cy5-dUTP (Cy5) and measures approximately 150 nucleotides (data not shown). Though it is a single nucleotide, the Cy5 modification is large, which could explain its delayed migration and detection.

Using an Agilent Bioanalyzer, typical indications of high quality, intact total RNA samples are electropherograms with flat baselines and a relatively flat valley between the two strong fluorescent rRNA peaks (Figure 1A, No RNase). An important note is that within the context of a high quality total RNA sample, the mRNA fluorescence is below detection. mRNA normally accounts for only 1–5% of a total RNA sample [9] and the rRNA peaks dominate the fluorescence of these samples. The undegraded total RNA sample shows distinct 18S and 28S rRNA subunit spikes, with a ratio of 1.85 ([28S]: [18S]). This ratio, along with the contours of the electropherogram, led to the conclusion that this was an undegraded, high quality RNA sample. Treatment with 1 ng ml-1 RNase degrades essentially all RNA present, as evidenced by its lack of 18S and 28S rRNA peaks (Figure 1D, 1 ng ml-1 RNase). Concentrations of RNase less than 1 ng ml-1 only partially degrade the RNA samples since 18S and 28S peaks are detectable but less than optimal (compare Figure 1A with 1B and 1C, No RNase with 100 ng ml-1 and 10 ng ml-1 RNase, respectively).

Fully degraded, partially degraded and intact RNA, generated by titrating varying amounts of RNase, were used to prepare samples for measuring Cy5 incorporation directly into the cDNA by reverse transcription of RNA by the Agilent 2100 Bioanalyzer. It should be noted, however, that RNase treatment can be unpredictable, since even slight deviations in temperature and timing of incubation can result in varying degrees of degradation. Over triplicate experiments, individual treatments could vary over 100% from day to day, however the overall trends do not change.

Cy5-dUTP was directly incorporated by reverse transcription into the intact (No RNase) sample as well as the three RNase treated samples. Since the RNA is hydrolyzed after reverse transcription, only the cDNA detectable. Analysis of the Cy5 signal obtained from an intact RNA sample (Figure 1E, pink trace), as measured by the Bioanalyzer, reveals a distribution of different transcript sizes. Along with a wide distribution of sizes, the overall fluorescence is much higher for the undegraded sample as compared to the RNase-treated samples (blue, red and green traces for 0.01, 0.1 and 1 ng ml-1 RNase, respectively, Figure 1E). The Cy5-labeled cDNA concentration, calculated by the Bioanalyzer assay as the area under the curve in Figure 1E, decreases approximately 65% after treatment with 1 ng ml-1 RNase and 30% with 0.1 ng ml-1 RNase. Treatment with 0.01 ng ml-1 RNase also depleted Cy5-labeled cDNA concentration, though only by about 25%. These Bioanalyzer results show considerable reductions in Cy5-labeled cDNA signal obtained, due to the degraded nature of the RNA samples.

**Figure 2:**
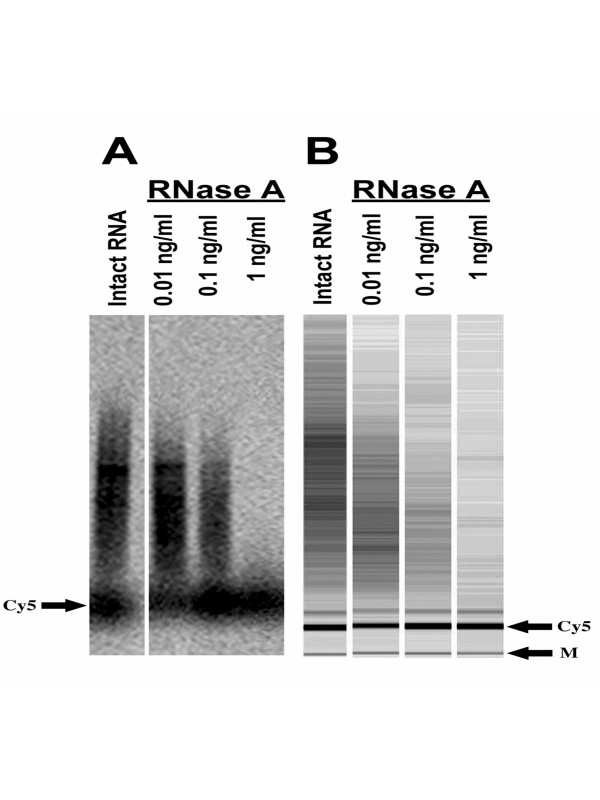
**Comparison of Bioanalyzer assay with conventional gel analysis. **A- cDNAs were separated based on size using a 1% agarose gel. Red fluorescence (650 long pass emission filter) was measured using a Storm phosphorimager and quantitated using IMAGEQuant. B- "Gel-like" images obtained from the Bioanalyzer are digital images rendered from conversion of electropherogram fluorescence traces using the Bioanalyzer software. Both images are the same sample from one representative experiment ran in parallel.

Gel electrophoresis, such as small scale poly-acrylamide analysis [10], has been used to determine characteristics of Cy5 incorporation. As shown in Figure 2, the phosphorimager scan of an agarose gel and the "gel-like image" obtained from the Bioanalyzer visually depict common trends. However, performing the gel electrophoresis was more sample- and time-consuming. Furthermore, quantitation was more subjective since measurement areas must be user-defined. The coefficients of variation, though intrinsically high because of the RNase treatment itself, were higher for the gel electrophoresis analysis.

Further validation of this assay came from analyzing RNA samples that had previously been labeled and hybridized, but provided poor quality expression data. These RNAs, based on Bioanalyzer electropherograms similar to that of the intact RNA in Figure 1A, appeared to be of high quality with 18S:28S peak ratios in excess of 1.8. To test the new assay, the labeling reactions were performed again and the labeled products produced were assessed on the Bioanalyzer and hybridized to the NIEHS Human ToxChip.

**Figure 3:**
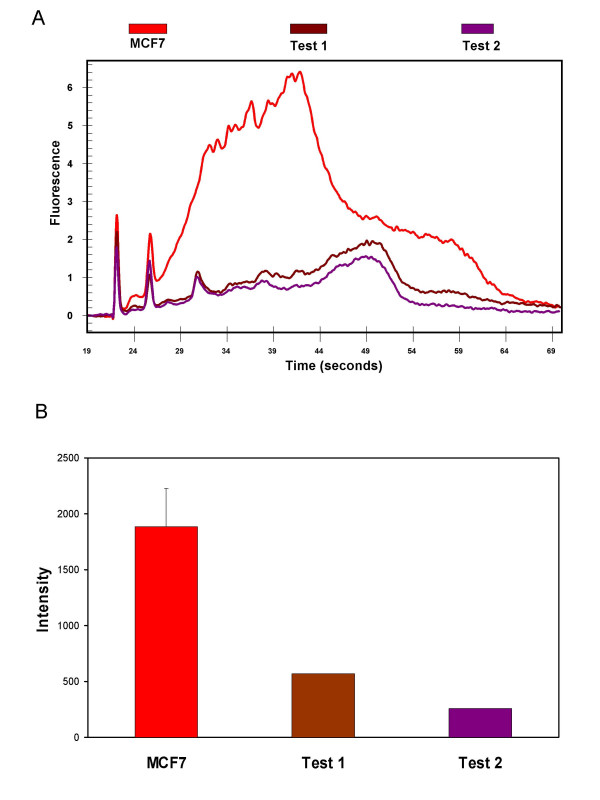
**Practical application of the Cy5-cDNA Bioanalyzer assay. (A) **Overlayed electropherogram image of Cy5-dUTP labeled cDNA obtained from control RNAs (MCF7 and HACAT, red and blue traces, respectively) and from test samples (Test #1 and Test #2, brown and purple traces respectively). First and second sharp peaks are as described in legend for Figure 1. **(B) **Average microarray intensity for MCF7, Test 1 and Test 2. Error bar is standard error from three arrays. Test samples were hybridized to only one microarray each.

As shown in Figure 3A, each of these test samples show a marked decrease in the total fluorescence measured by the Bioanalyzer as compared to the control samples, which were derived from RNA that had previously performed well in microarray labeling and hybridization procedures. Additionally, comparison of average array intensity for each of the test samples is dramatically reduced when compared with arrays hybridized with product from MCF7 intact RNAs (Figure 3B). We believe that there was possible genomic DNA contamination, as evidenced by non-migratory nucleotides in the wells of a formaldehyde gel (data not shown) It has been implicated that DNA contamination causes a decrease in efficiency of the labeling procedure [11], though other contaminants inherent to RNA extraction, such as phenol, or inherent to the RNA sample (high lipid or protein content) may also interfere. Though first thought to be of ideal quality for microarray, these human lung fibroblast test samples have been shown through the application of this novel assay to be less than optimal for use on a microarray chip. Had this assay been used prior to hybridization in previous experiments, the money and time spent in putting these direct labeled products on a chip would have been saved.

## Conclusion

From this novel Bioanalyzer assay, we are able to determine if a sample of questionable integrity is "chip worthy" or not. Selection criteria are subjective since the quality requirement will differ for different platforms. Determining the integrity of a sample could save an investigator a significant amount of capital resources (compare the tens of dollars that a Bioanalyzer chip costs to the hundreds of dollars in expenses for a microarray hybridization). The Agilent 2100 Bioanalyzer provides a cost-efficient way to determine an RNA, and the subsequent cDNA, sample's ability to provide high quality data (wide dynamic range) for a microarray chip. Though not meant as an absolute quantitative tool, trends in labeling efficiency can be assessed using this novel method. This assay can be used in place of conventional gel electrophoresis because it is more time and sample efficient, and it is considerably more cost effective than microarray hybridizations. In the future, we plan to use this assay to as a screening tool for direct labeled products prior to hybridization.

## Methods

Standard protocols can be accessed at .

### RNA preparation and degradation

Total RNA was prepared from logarithmically growing immortalized human mammary cells (MCF-7) using the RNeasy kit (Qiagen, Valencia, CA, USA) according to the manufacturer's instructions. RNA concentration and purity were estimated spectrophotometrically and the quality further assessed with the Agilent 2100 Bioanalyzer (Palo Alto, CA, USA). This intact RNA was split into aliquots and treated with varying concentrations of RNase A (0, 1, 0.1 and 0.01 ng ml-1). After incubation at 37°C for 10 minutes, RNA was analyzed for post-treatment quality assessment using the Bioanalyzer. The samples were loaded at 100 ng per well based on the original concentration values obtained by the spectrophotometer. The Agilent 2100 Bioanalyzer uses a 635 nm diode laser with an emission filter of 670–700 nm to detect fluorescence after dye intercalation into nucleic acids.

Total RNA was also prepared from human keratinocytes (Hacat) and human lung fibroblasts (16Lu) (American Type Culture Collection, Manassas, VA, USA) using the RNeasy kit (Qiagen, Valencia, CA, USA) according to the manufacturer's instructions. RNA concentration and purity were estimated spectrophotometrically and the quality further assessed with the Agilent 2100 Bioanalyzer (Palo Alto, CA, USA).

### Direct Cy5-dUTP incorporation for RNase-treated samples

Each total RNA sample (35 μg, post-RNase treatment) was labeled with direct incorporation of Cyanine 5 (Cy5)-conjugated dUTP (Amersham, Piscataway, NJ) by a reverse transcription reaction using the reverse transcriptase, SuperScript II (Invitrogen, Carlsbad, CA), and an oligo dT primer (Amersham, Piscataway, NJ). After reverse transcription, RNA was degraded by alkaline hydrolysis and excess dNTPs and enzymes from the reverse transcription reaction were removed using a modified version of Qiagen's QIAquick PCR Purification procedure (addition of a 35% guanidine HCl wash). This additional wash step occurs before the PE Buffer wash and uses the standard 750 μl volume for these columns. Following column washes, bound cDNA was eluted according to manufacturer's instructions.

### Direct Cy-dUTP incorporation for assay validation samples

Direct labelings (25 μg total RNA) by Cy3 and Cy5 were performed with two of the 16Lu RNAs being labeled with Cy3 and another two being labeled with Cy5. Additionally, MCF7-derived RNA as well as Hacat-derived RNA, both known to be of high quality, were labeled once per dye and are considered as controls. After the reverse transcription reactions were carried out, each Cy3/Cy5 pair was mixed together, cleaned and eluted using the Qiagen PCR Purification kit with modifications, as described above.

### Cy5 detection by the Agilent 2100 Bioanalyzer

For comparing the RNase-treated samples, 1 μl of the eluate was mixed with 5 μl 25% Sample Buffer (200 mM TAPS, 1 mM EDTA, pH 8.0) from the Agilent RNA 6000 Nano Kit, loaded onto an RNA LabChip (Agilent Technologies, Wilmington, DE) and detected with the Eukaryote Total RNA Nano Assay. Size distribution, concentration and overall amount of fluorescence were used for evaluation of dye incorporation. The detected fluorescence is the total combination of the intercalating dye and Cyanine 5. Comparisons are made between each of the RNase treated samples and the untreated control.

For the human lung fibroblast test samples, the Cy3 and Cy5 samples were mixed together in preparing for the hybridizations. We previously observed a lack of significant contribution to the overall fluorescence on the Bioanalyzer by Cy3-labeled samples (data not shown). Cy3 does not fluoresce in the range of the Bioanalyzer laser and any contribution to the outcome of the test would be minimal and yet consistent across all samples. One μl aliquots were analyzed as described above. Quality assessment was achieved by comparing the curves of the test samples to those of control samples.

### Gel electrophoresis

Five μl of each eluate was loaded onto a 1% agarose gel. The loading dye used in this experiment (25% glycerol, 0.1 M EDTA, 0.03% Bromophenol Blue) does not autofluoresce when measuring the red fluorescence and therefore did not interfere with measurement of the Cy5-labeled cDNA (data not shown). Red fluorescence (650 nm long pass emission filter) was detected using a Storm phosphorimager and quantified with IMAGEQuant (Amersham Biosciences, Piscataway, NJ).

### Microarray hybridizations and analyses

Fluorescently labeled test sample and MCF7 cDNAs were mixed with an SSC/SDS hybridization solution and hybridized to the microarray overnight in a 65°C water bath. The MCF7 samples were hybridized on different days than the test samples. A cDNA Human ToxChip [12], developed in-house at NIEHS, was used for hybridization experiments. A complete listing of the genes on this chip is available at the following website: . cDNA microarray chips were prepared as previously described [4,13].

The cDNA microarray chips were scanned with an Agilent Scanner (Agilent Technologies, Wilmington, DE) using laser excitation at 635 and 100% PMT sensitivity. The Agilent scanner is useful for these comparisons because of its ability to control laser variability, allowing data to be compared directly from day to day. The raw pixel intensity images were analyzed using the ArraySuite v2.0 extensions of the IPLab image processing software package (Scanalytics, Fairfax, VA). This program uses methods that were developed and previously described [14] to locate targets on the array, measure local background for each target and subtract it from the target intensity value. Data were calculated by taking the median of spot intensities over each chip

## List of abbreviations used

RNA Ribonucleic Acid

rRNA Ribosomal Ribonucleic Acid

mRNA Messenger Ribonucleic Acid

cDNA Complementary Deoxyribonucleic Acid

PCR Polymerase Chain Reaction

RT-PCR Reverse Transcription – Polymerase Chain Reaction

dNTPs deoxy-Nucleotide TriPhosphates

## Authors' contributions

SFG carried out the microarray analysis, performed most experiments, and wrote the paper

EKL provided scientific advice, performed traditional gel assay and recommended additional experiments

CJT conceived of the study, and participated in its design and coordination. All authors read and approved the final manuscript.
